# Osteopathic Manipulative Treatment as a Complementary and Integrative Approach for Cancer Supportive Care: A Systematic Review

**DOI:** 10.3390/cancers18121881

**Published:** 2026-06-09

**Authors:** Stuti Patel, Christopher J. Thimons, Hannah Steele, Misha Mathur, David Boesler, Anupam Bishayee

**Affiliations:** College of Osteopathic Medicine, Lake Erie College of Osteopathic Medicine, Bradenton, FL 34211, USA

**Keywords:** osteopathic manipulative treatment, cancer, pain, complementary and integrative medicine, supportive care

## Abstract

**Background**: Cancer is one of the leading causes of disability in both the United States and worldwide. Its high global prevalence is accompanied not only by the burden of the disease itself but also by the adverse effects of treatment, which can significantly diminish patients’ quality of life (QoL). Osteopathic manipulative treatment (OMT), an emerging complementary therapy, seems to show promise in the improvement in quality of life (QoL) in these patients. The purpose of this systematic review is to evaluate the effectiveness of OMT as a complementary and integrative therapy for the relief of symptoms and improved functional capabilities in cancer patients. **Methods**: The literature search was conducted in accordance with Preferred Reporting Items for Systematic Reviews and Meta-Analyses guidelines using PubMed, Scopus, Science Direct and Web of Science databases. A risk of bias analysis was conducted by evaluating the included randomized controlled trials to assess domains, such as randomization, intended intervention, incomplete outcome data, outcome measurement, and selective reporting. Between the 20 randomized controlled trials and observational studies, information regarding OMT in the treatment of general cancers, oral, pediatric, head and neck, gynecological, breast, and gastric cancers was collected. **Results:** Our analysis displayed a patient-reported increase in QoL, especially through the reduction in pain and lymphedema, as well as an increase in overall functional capabilities. **Conclusions**: Overall, the findings underscore the potential role of OMT as an adjunctive therapy for patients with a wide range of cancers, contributing to symptomatic management and enhanced QoL.

## Simple Summary

This systematic review evaluates the efficacy of osteopathic manipulative treatment (OMT) as a complementary and integrative approach for supportive care across a diverse range of cancer types including, pediatric, breast, gynecological, and head and neck cancers. Analysis of 20 included studies indicates that OMT is primarily associated with significant reductions in pain and lymphedema, as well as improved functional capabilities and range of motion. These studies underscore the potential role of OMT as an effective adjunctive therapy with limited contraindications. Despite these promising results for symptomatic management, further research is required to confirm the long-term efficacy and impact of OMT on quality of life in general.

## 1. Introduction

**Figure 1:**
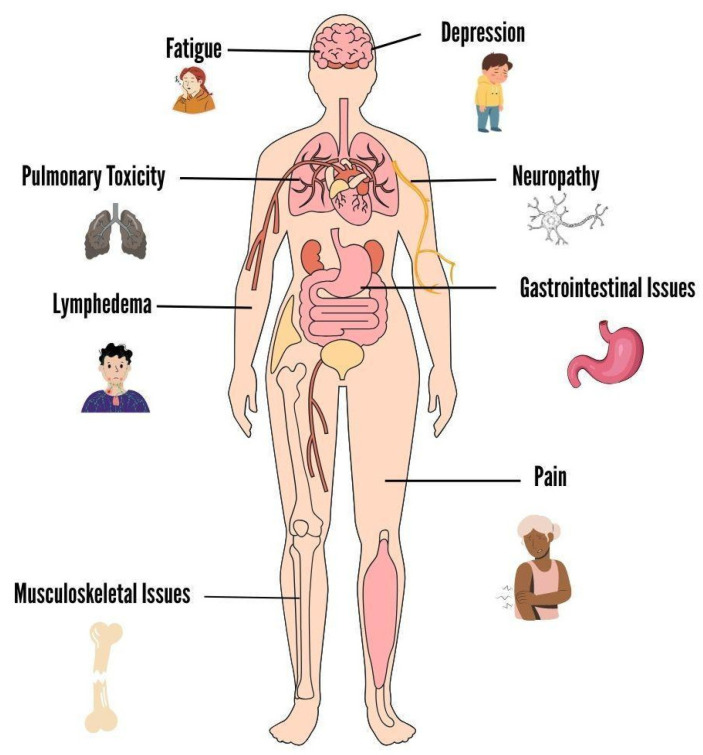
Common systemic symptoms and complications associated with cancer and associated treatments. Patients with cancer frequently experience a wide range of symptoms and complications, such as pain or lymphedema, that negatively affect both physical and psychological well-being. The figure displays many of the common symptoms and complications reported in these patients.

Cancer is one of the most deadly and rampant causes of morbidity and mortality in the world, affecting nearly one fifth of the population, and it is the cause of death of one twelfth of the population [1]. Current projections predict that in 2026 there will be 2,114,850 new cancer cases and 626,140 cancer deaths in the United States alone, corresponding to approximately 5794 new cases per day [2]. Cancer patients commonly have complaints of loss in functional ability, increased pain, and decreased quality of life. Alongside the burden of the cancer itself, various treatments, such as chemotherapy, radiation therapy, and surgical resections, are also associated with many detrimental side effects. These aggressive modalities of treatments have profound impacts on the patients, often causing functional limitations due to the dysregulation of the body’s homeostatic mechanisms [3]. Figure 1 displays the wide array of adverse effects that many cancer patients have noticed include pain, fatigue, neuropathy, lymphedema, depression, gastrointestinal impairments, musculoskeletal impairments, and pulmonary toxicity [4].

Osteopathic manipulative treatment (OMT) is one of the most utilized primary and adjunctive therapies to address pain, musculoskeletal deficits, and lymphatic drainage impairment [5]. OMT aims to increase systemic homeostasis and total patient well-being by applying hands-on manipulations to different body structures [6]. Various techniques that are often utilized in the musculoskeletal system by osteopathic physicians are muscle energy technique (MET), myofascial release (MR), balanced ligamentous tension, high velocity low amplitude (HVLA), and cranial osteopathy. On the other hand, techniques that are often performed to increase homeostatic function are rib raising, lymphatic pumps, and diaphragm doming [7].

Pain is one of the most common complaints of cancer patients, with nearly 44% of all cancer patients indicating the prevalence of some level of pain and an additional 30.6% of patients indicating moderate to severe levels [8]. The pain can be a result of many different mechanisms, such as bone metastasis, nerve compression, destruction of visceral structures, and therapeutic side effects. Despite the many causes of pain, the pain itself is often one of the most debilitating symptoms in these patients and causes an overall decrease in the quality of life (QoL) [9].

An adjunctive treatment, alongside traditional therapeutic regimens for cancer pain is the utilization of osteopathic manipulative techniques. These techniques, which are utilized by osteopathic physicians, focus on addressing somatic dysfunctions and alter or interrupt sympathetic outputs that correlate with increased levels of pain. While many different osteopathic manipulative techniques can address these physiological dysfunctions, MR is often used. In this technique, an osteopathic physician mobilizes the fascial structures of the patient’s affected area either with direct MR, which is towards a restricted boundary, or with indirect MR, which is towards an area of ease [6]. This technique causes an overall release in the tension associated with the affected fascia, increasing the overall tissue mobility and decreasing the associated somatic dysfunction. Rib raising may also be of use in the treatment of cancer-associated pain by decreasing the overall sympathetic input from the affected thoracic segments [10].

A decrease in functional capabilities, especially range of motion, is also a common complaint from cancer patients. This decrease in range of motion can be due to the burden of the cancer itself or the treatment. While many osteopathic techniques are utilized to increase range of motion, two of the most utilized are MET and HVLA [6]. Muscle energy is a technique that most commonly uses the Golgi tendon organ’s autogenic and reciprocal inhibition responses to stretch. Once the technique is utilized, the associated muscles will have an increase in overall mobility. The technique of HVLA is also a commonly utilized osteopathic technique to increase the range of motion and overcome the restriction of movement in patients in specific joints. This technique first requires the physician to identify a restriction of motion in a specific joint, then place the patient in a direct position at the boundary of that restriction, and assert a force that consists of high velocity and low amplitude [5]. The goals of the technique are to increase the overall range of motion, decrease the severity, or resolve the previously diagnosed dysfunction [6].

A lack of lymphatic drainage leading to central and peripheral edema is also a frequent complaint of patients with previous or current cancer diagnoses. Various osteopathic techniques focus on the treatment of lymphatic drainage through the body, such as lymphatic pumping, diaphragmatic doming, and opening of fascial inlets. The edema seen in cancer patients can be seen from different mechanisms ranging from decreased protein production in hepatic carcinomas to axillary node dissection to decrease the metastatic ability of breast cancer [11]. The use of these techniques is indicated for the mobilization of this excess and static lymphatic fluid, which is often responsible for these patients’ complaints and symptoms. In these patients, the doming technique will help strengthen the necessary diaphragmatic movements of the body, while the pumping and opening of the inlet will increase the overall lymphatic movement and drainage [12].

The benefits of OMT as an adjunct to cancer management and palliative care are evidenced by the existing literature. Brown et al. [13] elaborated on the importance of the osteopathic philosophy in cancer care by focusing on the person as a unit, the body’s ability to self-heal, and the interrelation between structure and function. Wojcik et al. [14] presented the theoretical application of osteopathy in oncology-specific palliative care, focusing only on the possibilities of OMT in oncological treatment and palliative care, but not studying its application. Murphy and Sokolof [15] discussed the use of OMT to correct somatic dysfunctions, which can improve cancer-related pain and symptoms. There are several prior reviews that have examined the usefulness of OMT for the treatment of cancer and palliative care. Fortin et al. [16] explored the possibility of incorporating OMT into treatment to reduce pain and psychological symptoms in women who have been diagnosed with breast cancer. Martone et al. [17] showed that the incorporation of OMT in cancer rehabilitation relieved pain and improved joint mobility, after the treatment of head, neck, and breast cancers. Yao et al. [18] summarized different integrative medicine modalities used in pediatric and young adults with cancer and found that the use of OMT resulted in a decrease in overall pain. Very recently, Wu et al. [19] evaluated the effectiveness of manipulative treatment, including massage, chiropractic, osteopathic medicine, and others, and indicated a reduction in cancer-associated pain compared to standard care. At least one systematic review and meta-analysis has been conducted to explore the effectiveness of OMT incorporation into the treatment of cancer. Lara-Palomo et al. [20] analyzed the effect of the myofascial therapy on pain and functionality of the upper extremities of breast cancer survivors based on eight randomized controlled trials. Although the investigators implied greater overall effects to support the intervention with myofascial therapy, the sub-group analysis found inconclusive results, indicating the need for further investigation to indicate any possible benefit.

While the previously mentioned literature has explored the impact of OMT on cancer-associated symptoms and side effects of current cancer treatments, each article focuses exclusively on a single type of cancer and its individualized complications, the potential of OMT incorporation, or the overall opinion around the incorporation of OMT into cancer care. Given the growing prevalence of cancer, there is an urgent need to explore interventions that can improve the QoL in a wide spectrum of cancers and patient populations. This systematic review examines the efficacy of OMT in patients diagnosed with cancer across a range of cancer types and treatment stages. This review also analyzes techniques, such as myofascial release, muscle energy, lymphatic drainage, and cranial approaches. These techniques are compared with standard care, sham treatment, or alternative physical therapies where a comparator was available to evaluate symptom-related outcomes, including pain, lymphedema, range of motion, functional capacity, and QoL.

## 2. Methods

**Figure 2:**
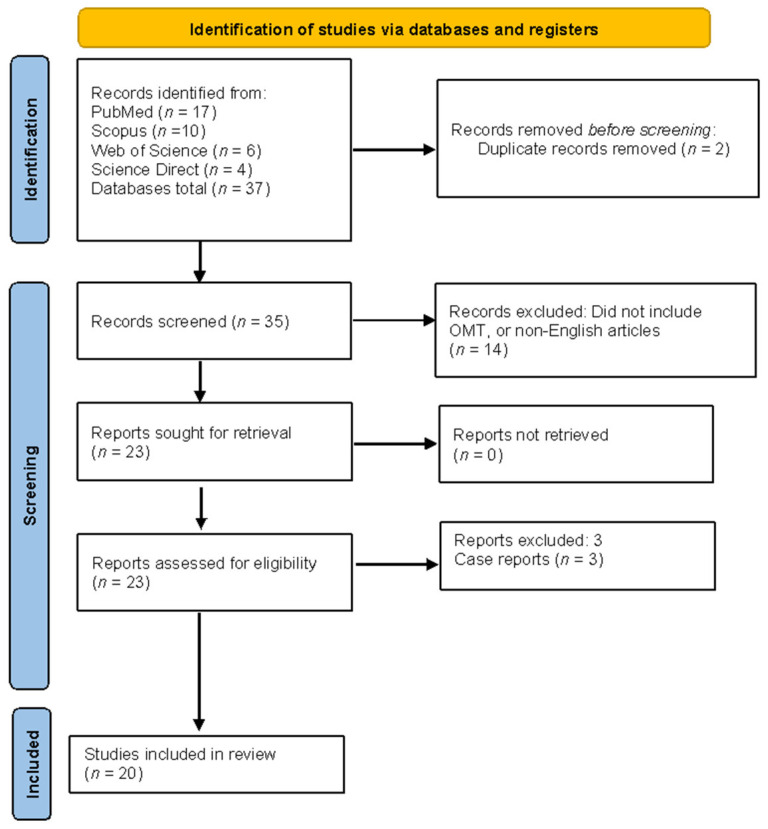
Preferred Reporting Items for Systematic Reviews and Meta-analyses (PRISMA) flowchart summarizing the selection process. The identification process refers to initial database search, and 37 articles were identified. The screening process consisted of primary author evaluation of articles based on inclusion and exclusion criteria. Following the screening process, 20 articles were included in the review.

A systematic search was conducted to identify research articles relevant to this study. The literature review followed the guidelines described by the Preferred Reporting Items for Systematic Reviews and Meta-Analyses (PRISMA), as outlined by Liberati et al. [21] and later updated by Page et al. [22]. The databases used for the search included PubMed, Scopus, Science Direct and Web of Science. The search window did not have restrictions on the publication year. The literature search occurred from August 2025 to March 2026 and was conducted by all authors. The final literature search in each database occurred on 13 March 2026. Keywords utilized in the search included osteopathy, osteopathic, cancer, palliative, osteopathic principles and practice, complementary, manipulative medicine, myofascial, osteopathic approach, and osteopathic manipulative treatment. Search queries included OMT AND Cancer, Osteopathic AND Cancer, Complementary AND Cancer. Field tags, filters and limits were not used in search queries. Reference lists, trial registries, and gray literature sources were not included in data collection. The articles were initially screened based on abstracts and keywords. Articles satisfying the initial screening were further evaluated based on full text analysis in a comprehensive evaluation of all texts by all authors to reach a consensus on final inclusion. Two duplicates were identified in this phase by manual review. The screening process is described in a PRISMA flow chart, as depicted in Figure 2. International Prospective Register of Systematic Reviews (PROSPERO) registration is generally not mandatory for conducting or publishing a systematic review, and accordingly, this review has not been registered with any such database. We acknowledge that limitations may arise as a result of lacking PROSPERO registration.

### 2.1. Inclusion Criteria

Inclusion was based on participant, intervention, comparator, and outcome (PICO) principles. These principles were utilized to maintain consistency and rigor. The protocol was established before conducting the review. Studies included patients who had recently undergone or were currently undergoing cancer treatment. Interventions in the studies included OMT protocols. Comparators included placebo OMT, alternative treatment methods, or medication. The primary outcomes of interest focused on objective and subjective measures of improvement following OMT. The specific outcomes depend on the specific details of each study given the heterogenous nature of this review. Further information about the PICO principles of the study is presented in Table A1. Additional inclusion criteria included measurable outcomes and in the English language.

### 2.2. Exclusion Criteria

Articles that did not focus on cancer patients, did not include OMT, not written in the English language and/or were case studies were excluded from review. Articles that met the exclusion criteria were discussed before exclusion from the study.

### 2.3. Data Synthesis

Initially 37 articles were identified after the database searches. Following the application of inclusion and exclusion criteria, 20 articles qualified for inclusion (Figure 2). Data from articles were identified and shared in a common location amongst authors. Due to the heterogeneous nature of the included studies, a meta-analysis was not conducted.

### 2.4. Review Process

All authors participated in the systematic extraction and analysis of data. Each of the three primary researchers independently focused on data extraction and analysis of specific cancer types. The extracted variables included the sample size, cancer type, OMT techniques utilized, treatment frequency and duration, outcomes, and adverse events. Following data extraction, the senior authors further evaluated the data extraction to ensure consistency and quality between the primary authors. The research team met several times to discuss items of disagreement to reach a consensus. Disagreements were solved by referring to the inclusion and exclusion criteria.

### 2.5. Risk of Bias Analysis

To assess the quality and validity of the included studies, the research team utilized the Risk of Bias for Randomized Control Trials (RoB 2) tool, created by the Cochrane Collaboration [23]. Our analysis systematically evaluated each article across five critical domains: bias arising from randomization processes, deviations from intended intervention, missing data, measurement of outcome, and selection of reported results. To maintain interrater reliability, authors included justifications for analysis to maintain consistency between primary researchers. The authors met to discuss the RoB2 results and discrepancies were resolved by engaging the senior authors.

## 3. Results

### 3.1. Various Cancers

Four studies explored associations between OMT and quality-of-life improvements, including reductions in pain and fatigue, in patients with various types of cancers. These studies encompassed randomized and nonrandomized clinical trials, qualitative analyses, and observational designs (Table 1).

Arienti et al. [24] conducted a nonrandomized controlled clinical trial on hospitalized geriatric oncology patients to investigate the effects of OMT on pain relief and quality in this population. In this study, each group (OMT and control) included 12 patients. Control group patients received only physiotherapy (PT), while those in the experimental group received both OMT and physiotherapy. A variety of OMT techniques performed by an osteopath were used including suboccipital decompression, dorsal and lumbar soft tissue, back and abdominal MR, rib raising, cervical spine soft tissue, and sacroiliac MR. At the start of the study, all patients were evaluated for pain intensity and QoL using the Numeric Rating Scale and the European Organization for Research and Treatment (EORTC) of Cancer Quality of Life Questionnaire Core 30 (QLQC30), respectively (Table 2). Although improvements in QoL were observed by the end of the study (QLQC30-SS: *p* = 0.058; QLQC30-GHS: *p* = 0.074; QLQC30-FD: *p* = 0.500), these changes, along with between-group comparisons of NRS and QoL using the Mann–Whitney test, were not statistically significant. However, reductions in pain intensity at week 2 (*p* = 0.004) and week 4 (*p* = 0.002) were statistically significant.

Steel et al. [25] conducted a qualitative study using semi-structured interviews of cancer patients who received osteopathic treatment alongside conventional cancer treatment. In this research, 16 patients were interviewed, and a grounded theory approach was used to analyze the transcripts. Grounded theory is an inductive qualitative approach in which researchers repeatedly review transcripts, break them into meaningful units, and code them—first openly and then axially—to let themes and theory emerge directly from the data. Analysis of the interview transcripts revealed that the positive health benefits of OMT extended beyond pain relief to include improvements in sleep problems, edema, and fatigue.

Gras et al. [26] investigated the efficacy of complementary and alternative medicines (CAMs) in cancer patients by conducting a prospective survey. The study aimed to collect information on the type, frequency of use, and outcome of CAMs. Overall, 200 of the 209 screened patients were included in the study. The patients reported using a variety of CAMs including osteopathy, homeopathy, healing touch, naturopathy, magnetism, healing touch, Chinese medicine, reflexology, hypnosis, and suction cups. Osteopathy was the most widely used, with 49.5% of patients reporting prior osteopathic treatment; however, osteopathy was mainly reported to be used for reasons unrelated to cancer (60.6%). The remaining patients who reported using OMT indicated that they did so either to prevent or manage side effects of cancer treatment (12.8%) or to improve overall well-being (27%).

In another study, Galeazzi et al. [27] conducted a randomized placebo-controlled trial to determine the effect of OMT on pain in cancer patients undergoing palliative care. Among the 75 patients selected for the study, 37 patients were placed in the OMT group, and 38 patients were placed in the sham group. Patients in the OMT group were treated by an osteopathic practitioner using cranial/craniosacral techniques, indirect MR, and soft tissue pressure. Patients were asked to complete a QLQ-C15-PAL quality-of-life questionnaire on the first and last days of the study and were asked to assess their pain every morning and evening using a self-administered visual analog scale (VAS) ranging from 0 to 100. The OMT group showed a significant drop in their average VAS scores starting at day 3 and continuing through days 4, 5, and 6. There was a 43.2% decrease in evening pain scores from D0 to D6 for patients in the OMT group versus only a 12.25% decrease for patients in the sham group. In the OMT group, there was a significant decrease in the number of patient-controlled analgesics doses delivered between D0 and D6. A significant difference in quality-of-life improvement between the OMT and sham groups (*p* = 0.000047) was also noted. The results suggest a trend toward more improvement with OMT.

Overall, the aforementioned studies indicate beneficial clinical outcomes following OMT in cancer patients. Both experimental studies showed significant improvements in pain and nonsignificant improvements in QoL. Furthermore, both qualitative studies reported that patients receiving OMT perceived meaningful benefits, including enhanced well-being and improved management of cancer-related treatment side effects such as pain, edema, and fatigue. OMT was not associated with any adverse effects in the experimental studies. It is important to note that the improvement in quality-of-life scores between treatment and control groups did not show statistically significant results, indicating that further research is needed in this area before a definitive conclusion can be drawn.

### 3.2. Breast Cancer

The effectiveness of OMT on breast cancer was discussed in six studies (Table 3). Major areas of focus in breast cancer treatment with OMT include lymphedema, range of motion, pain, and QoL.

Serra-Añó et al. [28] evaluated the effectiveness of MR on patients who underwent breast cancer surgery and radiotherapy. In a randomized control trial, thirteen subjects received MR, and eleven were assigned to a placebo manual lymphatic drainage group (PMLD). All participants received one 50 min session per week for 4 weeks. The subjects were evaluated using the visual analog scale (VAS) for pain, manual goniometer for range of motion, disabilities of the arm, shoulder, and hand scale (DASH) for shoulder functionality, the Functional Assessment of Cancer Therapy for breast cancer patients (FACT-B + 4) for QoL, and the Patient Health Questionaire-9 (PHQ-9) for depression. The evaluations occurred before treatment (T0), completion of treatment (T1), and one month after completion (T2). The MR group saw pain improvement at T1 and T2 (*p* < 0.05), range of motion (ROM) improvement for all motions except internal rotation (*p* < 0.05), and an increase in the QoL based on the FACT questionnaire at T1 and T2 (*p* < 0.05). These results suggest that MR can offer long-term functional improvements and pain relief following breast cancer surgery.

Lagrange et al. [29] conducted a randomized double-blind parallel controlled clinical trial that evaluated the effects of visceral osteopathy manipulation (VOM) on gastrointestinal side effects of chemotherapy and QoL using QLQ-C30. The study utilized a sample size of 94 women who had complete resection of breast cancer and were receiving a chemotherapy regimen of 5-fluorouracil 500 mg/m^2^, epirubicin 100 mg/m^2^, and cyclophosphamide 500 mg/m^2^ via intravenous injections. The patients all also had an antiemetic regimen that was standardized among the participants. Following the three cycles of chemotherapy, all the patients received either the control or treatment. The control group received superficial manipulation. There were 54 patients in the experimental group and 40 in the control group. The study did not find a decrease in the incidence of nausea, vomiting or constipation following VOM compared to the placebo; however, patients did report a higher QoL in the VOM group.

Castro-Martín et al. [30] evaluated a single myofascial induction (MI) treatment for the evaluation of the reduction in postmastectomy pain syndrome following breast cancer surgery. Twenty-one patients in the study received either MI or the placebo and later received the other treatment 4 weeks later. The study compared the range of motion, nerve sensitization, and pressure and pain sensitivity. Analysis of covariance (ANCOVA) revealed a significant improvement in range of motion in the affected arms and a significant improvement in the sensitivity of the affected ulnar nerves (*p* = 0.036). There was no significant improvement in the sensitivity of other nerves or in the pressure and pain sensitivity of any nerve (*p* > 0.05). The treatment was only conducted once, and this might be insufficient for broader clinical application.

Torres-Lacomba et al. [31] studied the impact of manual lymphatic drainage on the subjective pain index, active range of motion (AROM) of glenohumeral flexion and abduction, and perceived shoulder disability using the Oxford shoulder scale and health QoL using FACT-B through a randomized single-blind clinical trial. The study utilized a sample size of 96. The experimental group received manual lymphatic drainage, and the control group underwent standard arm exercises. Data were collected at the 3-week (A_1_), 3-month (A_2_), and 6-month periods (A_3_). Pain decreased in the experimental group compared to the control group at the A_1_ and A_2_ period (*p* < 0.001). AROM and perceived shoulder disability improved in the control group at A_1_ and A_3_ (*p* < 0.001).

Pereira de Godoy et al. [32] conducted a randomized, blind, crossover, and clinical trial to investigate the effects of manual lymphatic drainage in 66 breast cancer patients with lymphedema, specifically those whose affected limb volume exceeded the contralateral limb by at least 200 mL, using volumetric analysis via water displacement to measure the lymphedema. The lymphedema was measured both before treatment and following treatment. In the treatment setting, the patients received 1 h of manual lymphatic drainage and, in the control, they received 1 h of rest. The data were then analyzed using the paired *t*-test. A significant (*p* = 0.0001) reduction in limb volume was found for the manual lymphatic drainage group. 

Kim et al. [33] studied the impact of MR on the upper limb volume, pain, shoulder ROM, and chest mobility using DASH. Participant QoL was measured using FACT-B. The measured upper limb circumference was used to calculate the upper limb volume. The study used a crossover design, with a randomized sequence with a sample size of 30. The 30 participants were divided into two groups: 15 patients received the MR treatment first, and the other 15 received the control treatment of light pressure on skin. A *t*-test was used to analyze the results. The study found a statistically significant (*p* < 0.05) difference in limb volume and improvement in ROM (except for internal rotation), DASH and pain. 

Collectively, the evidence from these studies reveals the potential for OMT in breast cancer treatment. Myofascial release was shown to manage the impacts of breast cancer surgery by improving ROM and pain. Manual lymphatic drainage additionally managed the impacts of surgery by reducing the impact of lymphedema. VOM did not directly manage the side effects of treatment but did improve the QoL of patients.

### 3.3. Pediatric Cancers

Treatment with OMT in cases of pediatric cancers is the focus of this section (Table 4). The use of osteopathic medicine in these children is primarily for the relief from chemotherapy-related side effects, increased functional ability, range of motion, mobility, and pain.

Belsky et al. [34] conducted a cohort study where a total of 60 pediatric cancer patients were interviewed on their opinion on OMT as an adjunct therapy to relieve the disadvantageous side effects of chemotherapy. The interviews were conducted by a single interviewer who asked questions regarding the participants’ knowledge of OMT, the side effects experienced during chemotherapy, and their willingness to use OMT to help alleviate some of these unwanted side effects. The study analyzed data from patients older than 9 years old, their caregivers, physicians, and nurses. The consensus from the interviews displayed a shared belief of better management of chemotherapy side effects, and an interest in the incorporation of OMT. The limitations noted in this study are the small sample size and the fact that it was conducted at a single institution.

In a prospective observational cohort study conducted in Barbieri et al. [35], 104 children diagnosed with blood cancer were treated with muscle inhibition techniques, passive and active joint mobilization, deep skeletal muscle fascial techniques, and cardio-sacral approaches. The outcomes were measured with the use of the Goal Attainment Scaling (GAS), which analyzes changes in joint mobility, posture, visceral function, and cranial–sacral rhythm. The study concluded that OMT and precision-based exercises were well tolerated in the pediatric population and led to improved mobility and function. The results of this study displayed an increase in the range of motion in the spinal columns of 81%, extremities of 78%, chest and abdomen of 82%, and cranial rhythmic impulse of 76%. This study had only minor adverse effects, which were found to be reversible: three patients reported increased joint pain the day after treatment, and one patient reported increased nausea. These adverse effects were treated with re-tailored OMT or medication if needed.

In a retrospective cross-sectional study, Lüthi et. al. [36], examined 140 pediatric cancer patients at a Swiss pediatric Hematology–Oncology center. This study utilized online questionnaires filled out by caregivers to seek out information about the different types of complementary and alternative medicine that their children received during oncology treatments, including OMT. This study indicated that osteopathy was the most popular type of complementary and alternative therapy for cancer patients in the time interval of 5 years or more. No immediate limitations were noted in the study, but due to the data collection methods used, there may be potential for recall bias.

Research on the utility of osteopathic manipulative medicine in the pediatric population can also be seen in the prospective single-institution pilot study of Belsky and Brown [37]. In this study, hospitalized children and young adults were treated with osteopathic techniques such as myofascial release, muscle energy, balanced ligamentous tension, and visceral manipulation. The techniques were conducted, and then, the safety of the techniques was measured using the validated FACES pain scale immediately before and after OMT and by adverse event grading per Common Terminology Criteria for Adverse Events (CTCAE) 24 h posttreatment. The investigators concluded that hospitalized children and young adults with cancer who received OMT had no adverse events and noticed a decrease in overall pain. This work, through its utilization of patient-reported data following treatment, allows for possible recall bias. Moreover, the participants were 63.6% male with an average age of 18, which may direct the results of the study towards the older pediatric/young adult male population.

Throughout the literature, an interest in the utilization of osteopathic manipulative medicine as an adjunct to traditional cancer treatment in the pediatric population is observed. The aforementioned studies display how various osteopathic techniques can be used to relieve some of the symptoms associated with cancer and chemotherapeutic treatment, while improving overall function in the pediatric cancer population.

### 3.4. Head and Neck Cancers

Head and neck cancers include malignancies of the oral cavity, pharynx, larynx, nasal cavity, paranasal sinuses, and salivary glands. There are six studies that explored the effects of OMT on patients with these cancers (Table 5).

Parab and Pattanshetty [38] performed a randomized clinical trial to analyze the effectiveness of MR versus MET on trapezius spasm for pain, disability, ROM, and QoL in patients with head and neck cancer. The study involved 24 patients, with 12 receiving MR and 12 receiving MET. Both techniques were administered by a therapist once daily for 6 days. Baseline and post-treatment (day 6) evaluations included cervical and shoulder range of motion, QoL, the Neck Disability Index, and pressure pain threshold. Both groups experienced significant decreases in pain (*p* = 0.0001) and neck disability (*p* = 0.0022), along with notable improvements in cervical and shoulder range of motion (all *p* < 0.05). In contrast, the QoL did not show significant change following either intervention (all *p* > 0.05). Additionally, no meaningful differences were found between the groups, indicating the comparable effectiveness of the two techniques.

A randomized clinical trial conducted by Thomas et al. [39] examined the effects of MET versus active ROM exercises on shoulder function in head and neck cancer patients post modified radical neck dissection. In this study, 48 participants were included: 21 patients received active ROM exercises, and 25 received MET performed by a therapist. Subsequently, two participants dropped out due to the need for flap re-suturing and drain fixation in the middle of the study. Both groups were treated for 10 consecutive days, starting 3–5 days postoperatively. On the first and tenth days, pain intensity ratings, Global Rating of Change Scale (GRCS) scores, and shoulder ROM measurements were assessed. Significant improvements in shoulder ROM, pain, and GRCS scores (all *p* = 0.000) were observed in both groups. The MET group demonstrated significantly larger improvements in shoulder abduction (*p* = 0.026) and GRCS scores (*p* = 0.000) compared with the active ROM exercise group. However, changes in shoulder rotation (*p* = 0.08) and flexion (*p* = 0.533), as well as pain (*p* = 0.282), were not statistically significant in comparison to the active ROM exercise group.

In a crossover, blinded, and placebo-controlled study, Castro-Martín et al. [40] investigated the effectiveness of MR in improving outcomes for head and neck cancer survivors. The study included 22 participants who received both a single 30 min session of manual MR and a 30 min session of stimulated pulsated shortwave therapy (placebo). All interventions were performed by the same physical therapist. These sessions were separated by a 4-week washout period. Cervical and shoulder pain and ROM, maximum mouth opening, and cervical muscle function were measured before and after every session. Following the single MR session, significant improvements in cervical (*p* = 0.026) and affected-side shoulder pain (*p* = 0.001), maximum mouth opening (*p* < 0.004), cervical muscle function (*p* < 0.001), and cervical ROM (all *p* < 0.004) were noted. Additionally, most values for cervical and affected-side shoulder pain, maximum mouth opening, and cervical ROM were clinically meaningful. A significant reduction in cervical pain (*p* < 0.004) was also noted in the placebo group; however, the reduction did not cross the threshold for clinical meaningfulness. The single MR session did not seem to provide any improvement in the shoulder ROM.

A randomized controlled clinical trial conducted by Ortiz-Comino et al. [41] also aimed to determine the effects of MR on head and neck cancer survivors. It is important to note that all participants were free of metastasis or active cancer and had completed oncological treatment in the previous 6–24 months. In this study, 46 head and neck cancer survivors were enrolled, where 20 were placed in the MR group and 23 in the control group. MR was carried out by physiotherapists. AROM of the shoulder and cervical spine, maximal mouth opening, handgrip strength, cervical endurance, perceived physical fitness, and the presence of temporomandibular dysfunction on the first and last days of treatment were examined. Significant improvements with the MR protocol were observed in maximal mouth opening (*p* = 0.001), temporomandibular dysfunction (*p* = 0.016), cervical endurance (*p* = 0.023), cervical AROM (all *p* < 0.04), affected shoulder abduction (*p* = 0.001) and unaffected shoulder flexion (*p* = 0.007) and external rotation (*p* = 0.026). Other shoulder AROM (all *p* > 0.05), handgrip strength (affected: *p* = 0.565; unaffected: *p* = 0.906), and perceived physical fitness (all *p* > 0.05) showed no significant improvements. The cervical AROM and affected-shoulder abduction were the only outcomes with changes that surpassed the established threshold for clinical meaningfulness.

Tsai et al. [42] aimed to compare the effects of rehabilitation exercises versus manual lymphatic drainage (MLD) in patients with oral cavity cancer via a randomized single blind study. Twenty patients were placed in the rehabilitation group (control group) and 19 patients in the MLD group. MLD was carried out by a physical therapist. Clinical measures included the VAS, cervical and shoulder ROM, ultrasonography and face distance for lymphedema, and the Foldi and Miller lymphedema scales. Both groups showed significant improvement in cervical ROM, VAS pain scores, and internal and external rotation of the right shoulder at the end of the intervention period. The left lateral cervical flexion, however, was significantly more improved in the MLD group (*p* = 0.038) compared to that of the control group. Concerning the lymphedema evaluation, right facial distance showed significant improvement in both groups (*p* = 0.005), but there was no difference in the Foldi and Miller scores and in the left facial distance. The skin to bone distance (SBD) of the bilateral horizontal mandible and left ascending mandibular ramus was significantly improved in both groups; however, the SBD of the right ascending mandibular ramus was significantly improved only in the MLD group (*p* < 0.001).

All the aforementioned studies that investigated the effects of OMT on patients with a history of head and neck cancer reported reductions in pain and some level of improvement in shoulder or cervical ROM. OMT treatment was not associated with any adverse effects in any of the studies. Overall, OMT may be a relatively safe non-invasive therapeutic option that can be used in conjunction with other medical therapies to manage post-operative outcomes in patients with head and neck cancer. However, further research is needed to fully assess the long-term efficacy of OMT in the treatment of head and neck cancer patients.

### 3.5. Gynecological Cancers

Gynecological cancers are cancers of the ovaries, cervix, fallopian tubes, uterus, vagina and external female genitalia. One study was analyzed that focused on OMT during surgical post-operative treatment.

Ettorre et al. [43] studied the impact of OMT on the hospital length of stay, postoperative pain scores, opioid use, and time to return of bowel function. Fifty patients received OMT, but the exact type of OMT was not specified in the study. Another 50 patients with similar surgeries were in the control group. The study revealed that the length of stay, opioid use, time to return of bowel function and post-operative day 2 pain scores were all significantly decreased after therapy, and 90% of patients said they would recommend OMT to others doing the same surgery (Table 6).

Analysis of only one gynecological cancer study reveals that OMT can have a positive impact on patients with gynecological cancer; however, information is scarce to make a complete assessment on the effectiveness of OMT in gynecological cancers. Further studies should be conducted to identify the most effective treatment modalities using OMT and to continue evaluating the effectiveness of OMT in gynecological cancers.

## 4. Discussion

The purpose of this systematic review was to evaluate the current literature examining the use of OMT as a complementary and integrative therapy in patients with various types of cancer. Studies focused on multiple cancer populations, including general oncology patients, head and neck cancers, pediatric cancers, breast cancer, and gynecological cancers, were included. Across these studies, the efficacy of OMT was measured using QoL questionnaires, range of motion measurements, and pain scales. The included studies utilized a wide range of osteopathic techniques, including myofascial release, muscle energy techniques, cranial approaches, lymphatic drainage, visceral manipulation, and soft tissue therapies, reflecting the individualized nature of osteopathic care. These interventions were applied across diverse populations, with five studies originating from Spain, four from France, three from the United States, two each from India and Italy, and one each from Brazil, South Korea, Switzerland, and Taiwan. A total of fourteen clinical trials, three cohort studies, two prospective surveys, and one cross-sectional survey were reviewed.

In studies involving patients with various cancer types, OMT was most strongly associated with reductions in pain and improvements in patient reported well-being [24,25,26]. One study even suggested decreased reliance on analgesic medications following treatment [27].

While some studies reported improvements in quality-of-life measures, many did not reach statistical significance. Several studies nonetheless showed trends favoring patients receiving OMT, though these results should be interpreted cautiously due to their subjective nature. The qualitative findings nevertheless suggested potential patient-perceived benefits extending beyond pain relief to include improved sleep, reduced fatigue, and enhanced overall comfort [25]. Together, these findings suggest that OMT may provide meaningful symptomatic relief for patients experiencing both disease-related and treatment-related burdens. The use of noninvasive treatments, such as OMT, could be particularly helpful in minimizing pharmacologic use and thus reducing risk of dependence and medication-related adverse effects. However, larger controlled trials are needed to establish the relationship between OMT and reduction in pain.

Among head and neck cancer populations, OMT interventions produced consistent reductions in pain and notable improvements in cervical and shoulder range of motion. Several studies also demonstrated improvements in mouth opening, cervical muscle function, and functional recovery [38,39,40,41]. Although changes in the QoL were not uniformly significant, the functional and symptomatic improvements observed were clinically relevant for a population often affected by long-term physical impairment following oncologic treatment. The improvements observed suggest that OMT may help address post-surgical myofascial restrictions that contribute to long-term disability.

The pediatric oncology literature further supports the feasibility and safety of incorporating OMT into cancer care. These studies indicated improvements in mobility, functional outcomes, and pain [34,36]. Several studies also indicated strong interest among patients, caregivers, and healthcare providers in utilizing osteopathic care as an adjunct therapy [33]. Minor adverse effects were reported in only one study and were found to be reversible, while others reported no adverse events [34]. It is important to consider, however, that much of the available pediatric evidence is based on observational study designs, which limit causal inference and may introduce potential bias and confounding. These findings highlight OMT as a potentially valuable supportive therapy for children undergoing intensive oncologic treatments. Additionally, the interest among parents and providers suggests that OMT could be well accepted within pediatric oncology settings if further research was conducted to establish standardized treatment protocols.

In breast cancer patients, the strongest evidence supported the use of OMT for postoperative pain, lymphedema, and functional impairments. Myofascial release consistently improved range of motion, pain levels, and shoulder related disability, while manual lymphatic drainage significantly reduced limb volume associated with lymphedema [27,30,31,32]. Several studies also demonstrated improvements in quality-of-life following OMT interventions. Although visceral osteopathic manipulation did not significantly reduce chemotherapy-related gastrointestinal symptoms, patients reported improved overall QoL, suggesting broader supportive benefits beyond symptom specific outcomes [28]. The management of breast-cancer related lymphedema with manual lymphatic techniques suggests that OMT could be used to enhance postoperative recovery.

Evidence related to gynecological cancers remains very limited but promising. The single study demonstrated reductions in postoperative pain, opioid use, length of hospital stay, and time to return of bowel function among patients receiving OMT [42]. High patient satisfaction further supports the potential role of OMT in postoperative recovery, though additional research is required to better define effective treatment approaches and confirm efficacy. Reductions in opioid use and length of hospital stay may have important implications for improving patient safety and comfort. Importantly, as the available evidence is derived from a single non-randomized study, the results should be interpreted with caution due to potential bias and confounding and cannot be considered definitive.

**Figure 3:**
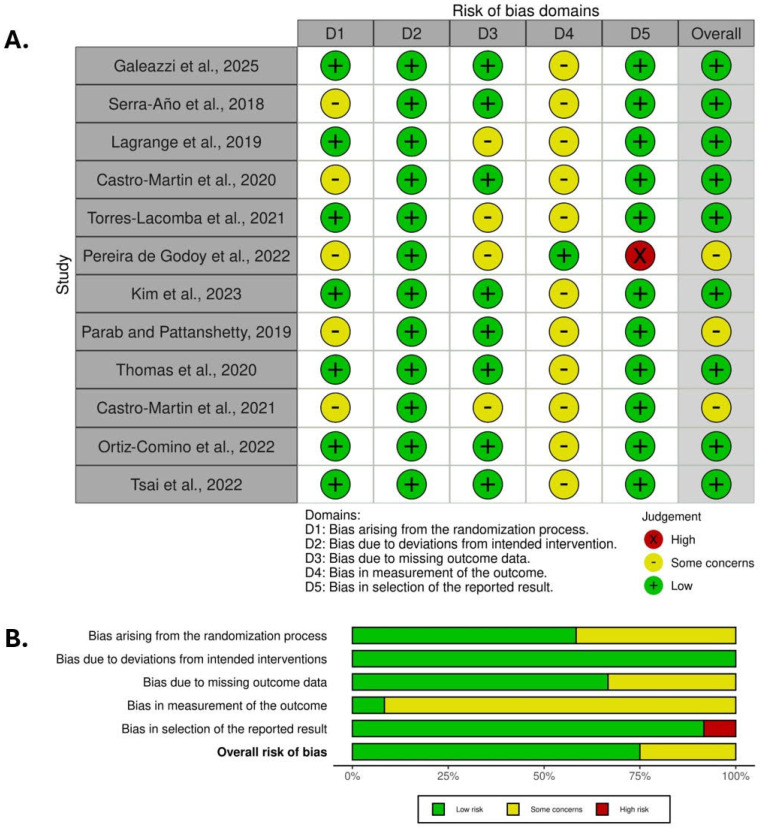
The risk of bias assessment for the 12 randomized controlled trials included in this review [27,28,29,30,31,32,33,38,39,40,41,42] was evaluated using the Risk of Bias in Randomized Control Trials tool (RoB 2). (**A**) Each study was evaluated using five domains: (D1) bias arising from the randomization process, (D2) bias due to deviations from intended intervention, (D3) bias due to missing outcome data, (D4) bias in measurement of the outcome, and (D5) bias in the selection of the reported result. Overall risk of bias is summarized in the last column. (**B**) The Summary of Risk of Bias Across Studies visually represents the percent of studies classified as either low risk (green), some concerns (yellow), or high risk (red) in each of the domains.

Although the results across cancer populations are largely positive, several methodological concerns limit the strength of the current evidence. Many studies were conducted with small sample sizes and brief treatment durations, which reduces the statistical power and restricts the ability to generalize findings to broader patient populations. In addition, the wide variation in disease/treatment stage, OMT techniques and treatment protocols makes it difficult to compare outcomes across studies or establish consistent clinical recommendations. The personalized approach central to osteopathic care further complicates efforts to standardize interventions for research purposes. Along with the personalized approach, OMT involves direct physical interaction between practitioner and patient; thus, participants are often aware that they are receiving treatment, and practitioners cannot be fully blinded to intervention delivery. Furthermore, Figure 3 indicates that 41.67% of studies were rated as having “some concerns” in Domain 1 (bias arising from the randomization process), largely due to small sample sizes and associated baseline imbalances. In addition, 33.33% of studies were rated as having “some concerns” in Domain 3 (bias due to missing outcome data), reflecting patient attrition and dropout during the study period. Moreover, 91.67% of included randomized controlled trials were rated as having “some concerns” in Domain 4 (bias in measurement of the outcome), largely due to the use of subjective outcome measures, including QoL surveys and pain scales. These endpoints rely on participant self-report and are inherently susceptible to reporting bias. The findings should therefore be interpreted with caution in light of the subjective nature of key outcomes. It should also be noted that randomized controlled trials provide the strongest evidence for causal inference, whereas observational studies are more vulnerable to confounding variables. These study designs therefore represent different levels of evidence and should not be interpreted as equivalent. Additionally, the possibility of publication bias may have influenced the available evidence, as studies with positive findings are more likely to be published. Language bias may also be present due to the inclusion of English-language publications only, which may exclude relevant studies published in other languages. Furthermore, ten of the twenty included studies were conducted in Europe, which may limit the generalizability of the findings to broader cancer populations and healthcare settings. Lastly, it is important to note the inherent bias of the placebo effect. Future studies that prioritize long-term follow-up would also be beneficial in establishing guidelines for use of OMT in comprehensive cancer care. Additionally, it may be beneficial for further investigations to include cost-effectiveness analyses to determine the broader healthcare impact of OMT being incorporated into oncology practices.

## 5. Conclusions

**Figure 4:**
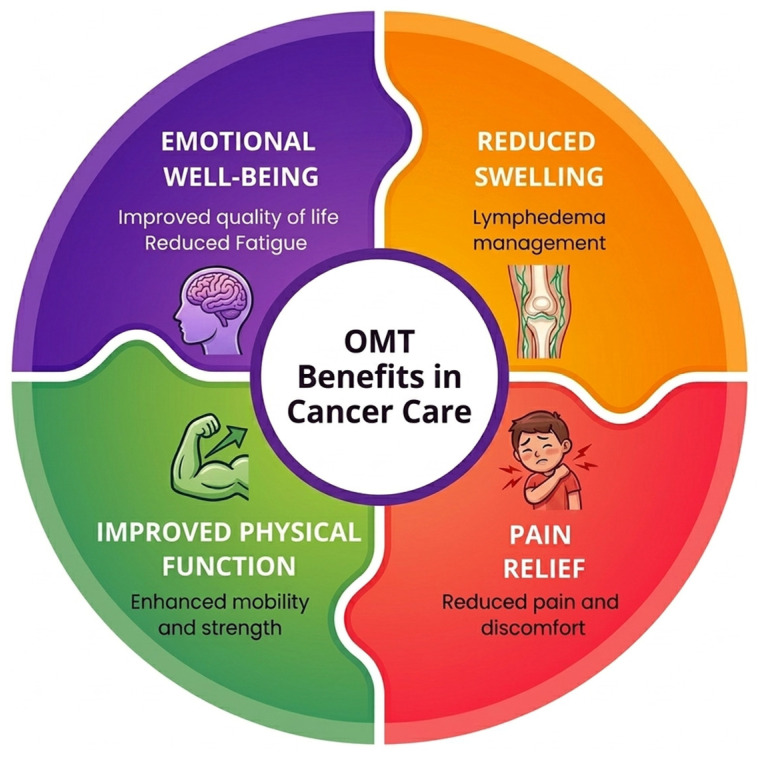
Overview of the beneficial effects of osteopathic manipulative treatment (OMT) as an adjunct to traditional cancer treatment. This image summarizes the key patient-reported and clinically measured outcomes described in the studies, ranging from measurable improvements in range of motion to enhanced QoL. The figure highlights OMT’s ability to provide both physiological and physical health benefits.

Overall, the current body of literature suggests that OMT is a potential safe noninvasive adjunct therapy that may provide meaningful improvements in symptom management and functional recovery among cancer patients; however, the current evidence remains insufficient to draw definite conclusions. Figure 4 depicts an overview of the beneficial effects of OMT as an adjunct to traditional cancer treatment. Improvements were most consistently seen in pain reduction, range of motion, functional recovery, and management of treatment related symptoms, with several studies also reporting enhancements in QoL. OMT was generally well tolerated, with minimal adverse effects reported. While these findings are encouraging, limitations such as small sample sizes, variability in treatment techniques, and reliance on subjective outcome measures indicate that the certainty of evidence amongst all outcomes remains limited, and stronger evidence is still needed before firm clinical recommendations can be made. The consistency of positive findings across diverse populations supports the need for continued investigation into OMT’s role as a complementary and integrative cancer therapy. Future research should prioritize adequately powered randomized controlled trials with standardized OMT protocols, as well as longer follow-up periods, to better establish the efficacy and improve the generalizability across various cancer populations. Future studies should further evaluate the safety and appropriate use of OMT in patients with conditions that may increase their vulnerability to complications, including bone metastases, thrombocytopenia, severe immunosuppression, postoperative wounds, radiotherapy-related tissue fragility, and advanced disease. Additional research is needed to better define contraindications, establish safety precautions, and identify which OMT techniques may be safest and most effective in these medically complex populations. In addition, economic evaluations are warranted to determine cost-effectiveness within oncology care settings.

## Figures and Tables

**Table 1:** Study design and characteristics of articles discussing general OMT use in various cancer patients.

Study Design	Sample Size	OMT Intervention	Control Group	Outcome	Reference
Nonrandomized controlled clinical trial	23	Physiotherapy and OMT: dorsal and lumbar soft tissue, rib raising, back and abdominal myofascial release, cervical spine soft tissue, suboccipital decompression, and sacroiliac myofascial release	Physiotherapy only	Significant improvement in pain relief and a nonsignificant improvement in QoL in hospitalized geriatric oncology patients.	Arienti et al., 2018 [24]
Qualitative prospective study	16	Nonspecific techniques	Not applicable	Supports resolution of pain, fatigue, and sleep problems.	Steel et al., 2018 [25]
Prospective survey	200	99 patients had OMT before	Not applicable	Use of CAMs led to patient satisfaction.	Gras et al., 2019 [26]
Randomized placebo-controlled clinical trial	75	Two 45 min OMT sessions: consisted mainly of indirect myofascial release, soft tissue pressure, and cranial and craniosacral techniques	Two sham sessions: applied manual contact on the patient in the same tested areas, but with no healing intention	Improved pain, QoL, and associated with a decrease in the number of PCA doses.	Galeazzi et al., 2025 [27]

Abbreviations: CAM, complementary and alternative medicine; OMT, osteopathic manipulative treatment; PCA, patient-controlled analgesia.

**Table 2:** Statistical tools utilized in studies discussing OMT use in cancer patients.

Control Group	Statistical Tools	References
General cancer		
Physiotherapy only	NRS and QLQC30 questionnaire	Arienti et al., 2018 [24]
Not applicable	Grounded theory approach	Steel et al., 2018 [25]
Not applicable	Repeated measures ANOVA and Scheffe post hoc test	Gras et al., 2019 [26]
Two sham sessions: applied manual contact on the patient in the same tested areas, but with no healing intention	QLQ-C15-PAL questionnaire, self-administered VAS, ANOVA, two-tailed tests	Galeazzi et al., 2025 [27]
Breast cancer		
Manual lymphatic drainage	Primary: VAS Secondary: ROM all movements of the shoulder, DASH, PHQ-9, FACT-B	Serra-Añó et al., 2019 [28]
Superficial manipulation	QoL Questionnaire QLQ-C30. Wilcoxon test, Chi2, Fisher’s exact	Lagrange et al., 2019 [29]
Unplugged pulsed short-wave therapy for 30 min	ULNT, ROM, ATOM, and ANCOVA	Castro-Martín et al., 2020 [30]
Standard arm exercises	Primary: Subjective pain index Secondary: AROM of glenohumeral flexion and abduction, perceived shoulder disability using OSS, HRQoL using FACT-B	Torres-Lacomba et al., 2022 [31]
One hour of rest	Volumetric analysis (water displacement) Paired *t*-test	Pereira de Godoy et al., 2022 [32]
Placebo MR for 30 min only used light pressure on skin surface	Upper limb volume, pain NRS, shoulder ROM, chest mobility, DASH, QoL (FACT-B). Paired *t*-test	Kim et al., 2023 [33]
Pediatric cancers		
None	Survey on OMT as a treatment to relieve chemo side effects	Belsky et al., 2021 [34]
None	GAS	Barbieri et al., 2021 [35]
None	Not specified	Lüthi et al., 2021 [36]
None	FACES pain scale and CTCAE 24 h post-OMT	Belsky and Brown, 2024 [37]
Head and neck cancers		
No control; the two techniques were compared against each other	Neck disability index, pressure pain threshold-pressure algometer, cervical and shoulder ROM and functional Assessment of Cancer Therapy—Head and Neck	Parab and Pattanshetty, 2019 [38]
Patients who had AROM exercises on shoulder function	ROM and pain intensity	Thomas et al., 2020 [39]
Patients who received mock pulsed shortwave therapy (placebo) for 30 min; this was decided by coin flipping	Cervical and shoulder pain with VAS and cervical ROM device and goniometer, MMO, and deep cervical flexor endurance test	Castro- Martín et al., 2021 [40]
Twenty-three patients who received the regular recommended regimen	MMO, TMD, cervical muscle endurance, AROM, handgrip strength, and physical fitness perception	Ortiz- Comino et al., 2022 [41]
Rehabilitation intervention	VAS pain score, neck and right shoulder ROM, *t*-test, 2-tailed chi-square test, generalized estimating equations	Tsai et al., 2022 [42]
Gynecologic cancers		
Control group of 50 hospital patients with similar surgeries	Comparison of the length of stay, postoperative pain scores, opioid use, and time to return of bowel function	Ettorre et al., 2023 [43]

Abbreviations: AROM, active range of motion; ATOMs, attitude towards osteopathic medicine; CTCAE, Common Terminology Criteria for Adverse Events; DASH, disability of arm, shoulder, and hand; GAS, Goal Attainment Scaling; HRQoL, health-related quality of life; FACT-B, functional assessment of cancer therapy—breast; MR, myofascial release; NRS, Numeric Rating Scale; MMO, maximal mouth opening; OMT, osteopathic manipulative treatment; OSS, Oxford Shoulder Score; PHQ-9, Patient Health Questionnaire-9; QLQ-C15-PAL, Quality of Life Questionnaire-Core 15-Palliative Care; QLQC30, Quality of Life Questionnaire Core 30; QoL, quality of life; ROM, range of motion; TMD, temporomandibular dysfunction; ULNTs, upper limb neural tension test; VAS, visual analog scale.

**Table 3:** Overview of studies related to OMT use in breast cancer patients.

Study Design	Sample Size	OMT Intervention	Control Group	Outcome	Reference
Randomized controlled trial	34	MR	Manual lymphatic drainage	Improvement in pain, ROM (except IR), and functionality at T1 and T2 compared to T0	Serra-Añó et al., 2019 [28]
Randomized double-blind parallel controlled clinical	94	VOM	Superficial manipulation	No decreased incidence of nausea, vomiting or constipation after undergoing VOM, but did report a higher QoL	Lagrange et al., 2019 [29]
Randomized, single-blind, and placebo-controlled crossover study (secondary analysis)	21	MR	Unplugged pulsed short-wave therapy for 30 min	Improvement in ROM for affected limb, and only ulnar nerve had significant improvement in sensitivity; there was no improvement in pressure pain sensitivity, but this may be because only one session of MR was performed, and more sessions may cause a statistically significant improvement	Castro-Martín et al., 2020 [30]
Randomized single-blinded clinical trial	96	MLD	Standard arm exercises	Reduction in lymphedema post mastectomy by using MLD Statistical difference with improvement in MLD group at end of trial and 3-month follow up.	Torres-Lacomba et al., 2022 [31]
Randomized, blind, and clinical trial	66	MLD	One hour of rest	Effective in reducing lymphedema	Pereira de Godoy et al., 2022 [32]
Crossover design, with randomized sequences	30	MR	Placebo MR for 30 min light pressure on skin surface	Primary: Statistically significant difference in limb volume Secondary: Improvement in ROM (except IR), DASH, pain	Kim et al., 2023 [33]

Abbreviations: MR, myofascial release; MLD, manual lymph draining; maximal mouth opening; OMT, osteopathic manipulative treatment; QoL, quality of life; ROM, range of motion; VOM, visceral osteopathy.

**Table 4:** Study design and characteristics of studies on OMT use in pediatric cancer patients.

Study Design	Sample Size	OMT Intervention	Control Group	Outcome	Reference
Three separate cohort studies	60 (20 patients each study)	None: this was based on interviews	None	Pediatric oncology clinicians, caregivers, and patients reported a need for better management of chemotherapy associated side effects and an interest in utilizing OMT.	Belsky et al., 2021 [34]
Prospective observational cohort study	104	Muscle inhibition techniques, joint passive and active mobilization, deep skeletal muscle fascial techniques, and cranio-sacral approaches	None	OMT with precision-based exercise was well tolerated and led to improved mobility and function in most children and adolescents with blood cancers.	Barbieri et al., 2021 [35]
Retrospective cross-sectional study	140	Not specified	None	Osteopathy was the most popular CAM ≥5 years after the end of oncological treatment.	Lüthi et al., 2021 [36]
Prospective single-institution pilot study	11	MR, MET, balanced ligamentous tension, and visceral manipulation	None	Hospitalized children and AYAs with cancer received OMT safely with decreased pain in their reported somatic dysfunction(s).	Belsky and Brown, 2024 [37]

Abbreviations: AYAs, adolescents and young adults; CAM, complementary and alternative medicine; MET, muscle energy technique; MR, myofascial release; OMT, osteopathic manipulative treatment.

**Table 5:** Study design and characteristics of studies on OMT use in head and neck cancer patients.

Study Design	Sample Size	OMT Intervention	Control Group	Outcome	Reference
Randomized clinical trial	24	MR and MET	No control; the two techniques were compared against each other	Reduction in pain and neck disability and improved neck and shoulder ROM.	Parab and Pattanshetty, 2019 [38]
Randomized clinical trial	48	MET	Patients who had AROM exercises on shoulder function	This study showed that both METs and AROM exercises were effective in improving shoulder range of motion, function and reducing pain, but muscle energy was more effective.	Thomas et al., 2020 [39]
Crossover, blinded, and placebo-controlled study	22	MR	Patients who received mock pulsed shortwave therapy (placebo) for 30 min; this was decided by coin flipping	MR improved pain, cervical ROM, MMO, and cervical muscle function, but not shoulder ROM, in survivors of HNC.	Castro- Martín et al., 2021 [40]
Randomized controlled trial	46	MR	23 patients who received the regular recommended regimen	MR improves MMO, TMD, cervical function (endurance and AROM), affected shoulder abduction, and unaffected shoulder flexion, and external rotation AROM in the sHNC.	Ortiz- Comino et al., 2022 [41]
Randomized single blind study	39	MLD and rehabilitation intervention	Rehabilitation intervention	The VAS pain score, ROM of the neck, and internal and external rotation of the right shoulder significantly improved.	Tsai et al., 2022 [42]

Abbreviations: AROM, active range of motion; HNC, head and neck cancer; MET; muscle energy technique; MR, myofascial release; MLD, manual lymph draining; MMO, maximal mouth opening; OMT, osteopathic manipulative treatment; ROM, range of motion; sHNC, secondary head and neck cancer; TMD, temporomandibular dysfunction; VAS, visual analog scale.

**Table 6:** Study design and characteristics of studies presenting OMT use in gynecologic cancers.

Study Design	Sample Size	OMT Intervention	Control Group	Outcome	Reference
Retrospective cohort study	50	Not specified	Control group of 50 hospital patients with similar surgeries	Length of stay, opioid use, time to return of bowel function, and postoperative day 2 pain scores were all significantly decreased after therapy. In total, 90% of patients said they would recommend OMT to others doing the same surgery.	Ettorre et al., 2023 [43]

Abbreviations: OMT, osteopathic manipulative treatment.

## Data Availability

Data are available on request from the authors.

## Abbreviations

ANCOVA	Analysis of covariance
AROM	Active range of motion
CAMs	Complementary and alternative medicines
CTCAE	Common Terminology Criteria for Adverse Events
DASH	Disabilities of the arm, shoulder, and hand
EORTC	European Organization for Research and Treatment
FACT-B	Functional Assessment of Cancer Therapy for breast cancer patients
GAS	Goal Attainment Scaling
GRCS	Global Rating of Change Scale
HVLA	High velocity low amplitude
MET	Muscle energy technique
MR	Myofascial release
MLD	Manual lymphatic drainage
OMT	Osteopathic manipulative treatment
PHQ-9	Patient Health Questionaire-9
PMLD	Placebo manual lymphatic drainage group
PRISMA	Preferred Reporting Items for Systematic Reviews and Meta-Analyses
PT	Physiotherapy
QLQC30	Cancer Quality of Life Questionnaire Core 30
QoL	Quality of Life
RoB	Risk of bias
ROM	Range of motion
SBD	Skin to bone distance
VAS	Visual analog scale
VOM	Visceral osteopathy manipulation

## Appendix A

**Table A1:** An outline of PICO protocols established for this article.

Population	Patients recently or currently undergoing treatment of a malignant condition.
Intervention	Osteopathic manipulative treatment through various modalities (myofascial release, muscle energy, lymphatic techniques, etc.)
Comparator	Other physical manipulation and treatments (physiotherapy alone, superficial treatment, sham treatment) and standard of care without osteopathic manipulative medicine
Outcomes	Quantitative improvement in quality of life or symptoms based on cancer and osteopathic manipulative treatment specifies
Study design	Clinical trials or prospective or retrospective cohort studies. Studies need to include human subjects. Exclusions include case studies, reviews, articles not written in the English language, or no osteopathic manipulative treatment.
